# Infection control for safety and quality

**Published:** 2021-07-20

**Authors:** Nicola C Gordon

**Affiliations:** 1 Clinical Microbiologist/Infectious Diseases Physician: Imperial College Healthcare NHS Trust, London, United Kingdom.

**Health care-associated infections can be painful, potentially blinding, and even life threatening. Infection prevention and control is therefore a vital part of caring for our patients.**

When a patient comes for medical treatment, they should not end up in a worse condition than when they arrived. Infection prevention and control (IPC) is the aspect of health care which aims to ensure that patients do not contract infections as a result of attending a health care facility for assessment, examination, or treatment. These are known as health care-associated infections. The basic principles of infection control have been known for centuries, but prevention of health care-associated infections is still a major challenge. Even though these infections are less common in eye care, they may result in vision loss and care should be taken to prevent them.

## Health care-associated infections in eye health

In eye health, the main health care-associated infections are:

- Acute conjunctivitis
- Endophthalmitis
- Respiratory tract infection.

**Figure:**
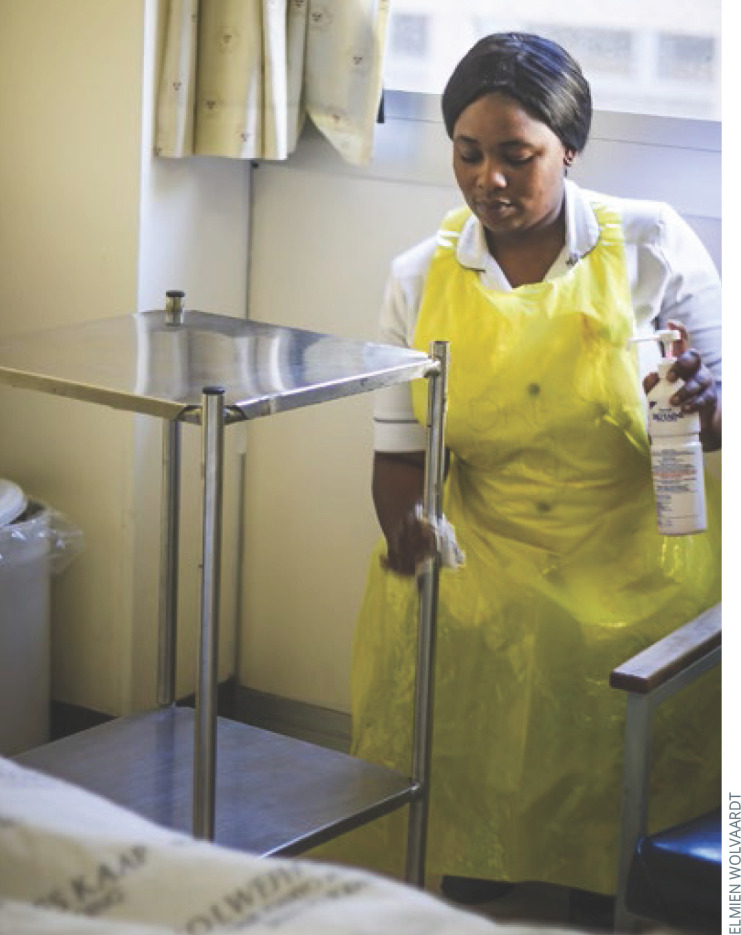
Disinfection of trolleys and trays is important before beginning any eye procedure, including changing an eye dressing. For invasive procedures, surgical asepsis is required, a strict process which uses maximal sterile barriers such as sterile drapes, gowns, and gloves. **SOUTH AFRICA**

Acute conjunctivitis is frequently caused by adenovirus, which is highly transmissible and can be acquired from contaminated equipment, including trial lenses and frames, or health care workers’ hands. Large outbreaks have been reported, and the impact can be very significant on eye services as health care workers may also be affected, resulting in sore eyes, blurred vision, and the need to take time off work.

Health care-associated endophthalmitis is usually due to bacterial infection, resulting from contaminated equipment or instruments, contaminated liquids such as eye drops or intravitreal injections, or poor surgical or clinical technique that introduces bacteria from the patient’s own skin or from the hands of the health care worker, usually after cataract surgery or intravitreal injection. Although the frequency of endophthalmitis is low, it is potentially devastating for the patient.

Respiratory tract infections due to viruses such as influenza, rhinovirus, and coronavirus may be transmitted more commonly in eye health than in, say, general practice. This is because eye care equipment and instruments (such as slit lamps) come into close contact with the patient’s face and can easily become contaminated with respiratory tract viruses that, in turn, may be transmitted to the next patient. Health care workers also work in very close proximity to patients when carrying out eye examinations or procedures and are therefore at risk of acquiring and transmitting these infections. This has become a more serious problem during the COVID-19 pandemic, and specific measures to reduce the risk of COVID-19 transmission in eye care were discussed in a previous issue of this journal.

## Reducing the risk to patients

Standard precautions relevant to reducing the risk of health care-associated infections in eye health are:

- Hand hygiene
- Environmental cleaning
- Safe reprocessing of reusable equipment and instruments
- Respiratory hygiene and cough etiquette
- Aseptic non-touch technique.

**Hand hygiene** can be achieved by washing with soap and water or, where water is not readily available, by using alcohol-based gels/sanitisers. Be aware that alcohol-based gels/sanitisers may be irritant to the eyes so, for contact procedures, soap and water is preferred.

Clinics and treatment rooms, including all surfaces, windows, doors, and fittings, should be **cleaned** regularly. Depending on the type of surface, alcohol wipes, alcohol solutions, or sodium hypochlorite solutions are generally recommended. Take extra care when cleaning surfaces which may be in contact with patients, such as chin and head rests or hand-held Snellen charts.

**Reprocessing** refers to the cleaning, disinfection, and/or sterilisation of reusable devices. The specific processes depend on the instruments and the manufacturer’s instructions should be followed, as well as any local policies. If it is practical and affordable, use single-use instruments and equipment instead and dispose of them safely.

**Figure 1:**
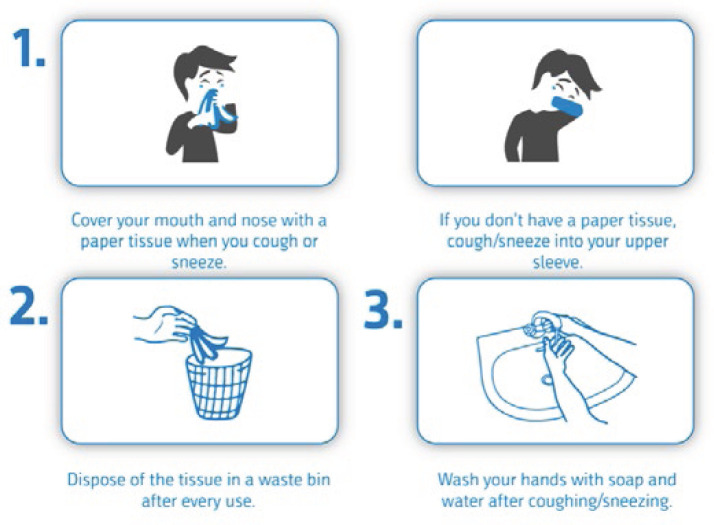
An infographic on respiratory hygiene and cough etiquette prepared by the Government of Slovenia

**Respiratory hygiene and cough etiquette** refers to the guidance given to patients and staff members about how to reduce the risk of viral transmission via coughs and sneezes (see Figure 1).

An **aseptic non-touch technique** should be used for procedures where applicable. For operative procedures, good aseptic surgical technique, including the use of sterile drapes, gowns, and gloves, is particularly important for preventing infections introduced as a result of contamination by the patient’s own skin flora. Reducing the use of multi-dose eye drop vials during examination – and using single-dose vials instead, where available – will also prevent transmission of infection between patients.

## Reducing the risk to health care workers

Health care workers examining and treating eye diseases may be exposed to infectious agents via respiratory droplets, tears, or blood. Practising good infection prevention and control, such as hand hygiene, use of personal protective equipment (PPE), safe use and disposal of sharps, and correct waste management will also reduce the risk of exposure.

**Hand hygiene** protects staff members from eye and respiratory tract infections acquired from patients. This risk is also reduced by using **personal protective equipment** (PPE), such as gloves, masks, protective eye wear, and face shields. Use of respiratory protection (masks and face shields) has become especially important during the COVID-19 pandemic.

**Sharps** pose a direct risk to the health care worker, but they also pose a risk to other staff members, such as cleaners. They must be properly disposed of in a sharps container, such as a box or tin.

All health workers must be aware of the local protocol for needle stick injuries, including assessment for post-exposure prophylaxis and following up with occupational health. Other contaminated waste should also be disposed of safely, for everyone’s protection.

**What does a safe and effective infection prevention and control programme look like?**

To improve the safety of patients and staff members, all health care facilities should have an infection prevention and control programme which takes account of the specific risks and infections that may occur.

**Figure 2:**
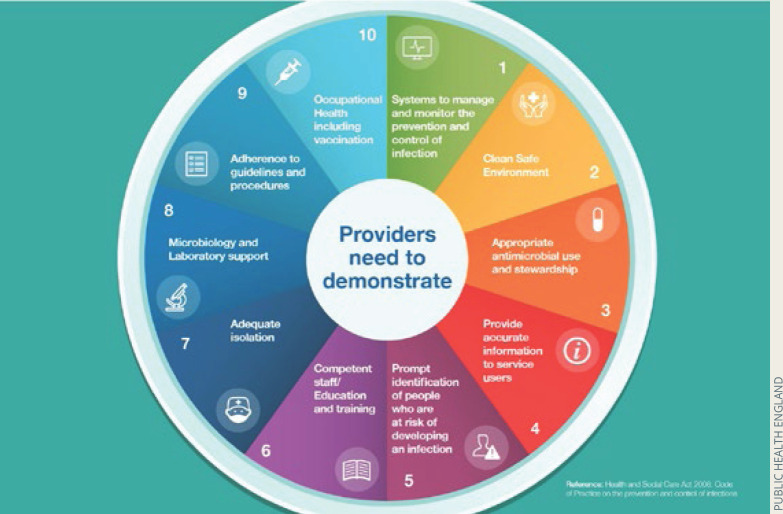
Infection prevention and control programmes have multiple components

Infection prevention and control programmes have multiple components (Figure 2), including:

- Systems to manage and monitor the prevention and control of infection
- A clean and safe environment
- Appropriate use of antimicrobials, including antibiotics
- Providing accurate information to patients and staff members
- Prompt identification of people who are at risk of developing an infection
- Competent staff (education and training)
- Adequate isolation
- Microbiology and laboratory support
- Adherence to guidelines and procedures
- Occupational health (including vaccination of staff members).

**Roles and responsibilities**

Although prevention of harm to patients through good infection prevention and control is everyone’s responsibility, infection control programmes should ideally be overseen by an **infection control team** comprising a senior nurse, an infection control doctor (usually a clinical microbiologist, infectious disease specialist, or hospital epidemiologist), and nurses or infection control practitioners with appropriate qualifications and experience. The infection control team is responsible for developing locally relevant infection control policies, including guidelines and standard operating procedures (SOPs). The team should also supervise training, surveillance and monitoring, and audit, and should report regularly to the hospital/institution management team or administration. The team should make themselves available on site, so that staff members can easily ask for advice about infection control matters. The infection control team should be supported by a multidisciplinary **infection control committee,** which makes decisions at institutional level, reviews any incidents or potential outbreaks, and supports the team to carry out wider organisational change where this is needed. The composition of the infection control committee will depend on the size of the institution, but should include managers, administrators, clinical and nursing leads, representatives from the different specialties (so that the programme takes into account specialty-specific needs) and representatives from estates, sterilisation services, and cleaning services. The committee should meet at regular intervals, usually 3 or 4 times a year, to review and discuss reports from the team and raise any concerns from their own departments. The committee should ensure that the infection control team have the necessary resources to run the programme by identifying priorities and needs and reviewing budgets accordingly. For example, the infection control team may identify an increased number of infections in patients undergoing surgical procedures and find that it is due to inadequate sterilisation of equipment. The team will present their report to the infection control committee, who discuss the findings and decide what action to take (e.g. repairing or replacing the equipment).

The team should have access to a **clinical microbiologist** for additional advice. Larger hospitals may have their own clinical microbiologist on site, in which case they will usually be part of the infection control team. Otherwise, a single microbiologist may cover several sites and provide on-call cover for treatment questions or outbreak control.

An effective infection prevention and control programme also involves ongoing monitoring and audit to ensure that the required standards are met. The infection control team should oversee the monitoring and audit programme and should present the results to the infection control committee so that actions can be taken where necessary.

## Conclusion

Good infection prevention and control is vital for reducing health care-associated infections and improving patient outcomes. This is particularly important with the emergence of antimicrobial resistance, as health care-associated infections are more likely to be multi-drug resistant and therefore difficult to treat. The constant evolution of microorganisms means that perfect infection control and prevention is unlikely; however, it is an ideal we must strive for. Hopefully, one of the lessons that will come out of the COVID-19 pandemic will be to pay more heed to routine infection prevention and control hygiene measures.

The multidisciplinary nature of the infection control committee is an important reminder that everyone has a responsibility to reduce health care-associated infections and prevent harm to patients. The role of the infection prevention and control team is to help all staff members to achieve this.
